# Influence of extraction methods on antioxidant and antimicrobial properties of essential oil from *Thymua danesis* subsp*. Lancifolius*

**DOI:** 10.1002/fsn3.269

**Published:** 2015-08-31

**Authors:** Yousef Tavakolpour, Marzieh Moosavi‐Nasab, Mehrdad Niakousari, Soroush Haghighi‐Manesh

**Affiliations:** ^1^ Department of Food Science and TechnologyCollege of AgricultureShiraz UniversityShirazIran; ^2^ Seafood Processing Research GroupCollege of AgricultureShiraz UniversityShirazIran; ^3^ Nanotechnology InstituteShiraz UniversityShirazIran; ^4^ Department of Food Science and TechnologySchool of AgricultureTarbiat Modares UniversityTehranIran

**Keywords:** Antimicrobial, antioxidant, essential oil, extraction, *Thymua danesis*

## Abstract

The essential oil (EO) from dried ground powder leaves and stems of *Thymua danesis* was extracted using hydrodistillation (HD), ohmic extraction (OE), ultrasound‐assisted HD and ultrasound‐assisted OE methods. Then, the antioxidant, antimicrobial, and sensory properties of the EO were investigated both in vitro and in food systems. Thyme EO extracted by ultrasound‐assisted HD method had promising antibacterial activities against *Escherichia coli* and *Staphylococcus aureus* and had the best antioxidant properties when tested in vitro. In food systems, higher concentrations of the EO were needed to exert similar antibacterial and antioxidant effects. Furthermore, thyme EO added yogurt and drink yogurt revealed better sensory properties than the control and fresh samples. Essential oil from *Thymua danesis* has a good potential to be used as an antioxidant, antimicrobial, and flavoring agent in food systems and the extraction method effects on the antioxidant and antimicrobial properties of the thyme extract.

## Introduction

Oxidation of lipids, which occurs during raw material storage, processing, heat treatment, and further storage of final products is one of the major reasons for deterioration of food products during processing and storage (Tomaino et al. 2005). On the other hand, it has been estimated that as many as 30% of people in industrialized countries suffer from a food borne disease each year. Therefore, investigation of new methods for reduction in foodborne pathogens and lipid oxidation of food products is needed. At the same time, there is an increasing trend of “green” consumerism, with lower side effects on consumers and environments (Burt 2004).

Plant EOs have been long recognized for their antibacterial, antifungal, antiviral, insecticidal, and antioxidant properties. They are widely used in medicine and the food industry for these purposes (Khosravi et al. 2009; Bassolé and Juliani 2012). Antioxidants are the compounds that, when added to lipid‐containing foods, can increase shelf life by retarding the process of lipid oxidation (Goli et al. 2005). Synthetic antioxidants have been used in food industry since the late 1940s. However, some physical properties of synthetic antioxidants, such as their instability at elevated temperatures, strict legislation on the use of synthetic food additives and consumer preferences have shifted the attention of manufacturers from synthetic to natural antioxidants from spices and herbs (Dapkevicius et al. 1998). Especially worthy of note are spices and herbs used for many years to enhance the sensory features of food (Tomaino et al. 2005).

The aim of this study was to compare the effect of four different extraction methods on antioxidant and antimicrobial properties of EO from *Thymua danesis* subsp. *Lancifolius* when tested in vitro and to investigate its antioxidant, antimicrobial, and sensory properties in food systems.

## Experimental

### Materials

*Thymua danesis* subsp. *Lancifolius* (Herbarium No. 23271) was collected from Fars province and verified by the Institute of Medicinal Plants, Shiraz, Iran. Sodium thiosulfate, sodium sulfate, methanol, acetic acid, chloroform, and hexane were all from Merck (Darmstadt, Germany).

### Plant sample preparation

At full flowering stage, the aerial parts of the plant (contained 900 g kg^−1^ leaves and 100 g kg^−1^ fine stems) were air dried at ambient temperature in the shade and powdered.

### Yogurt and drink yogurt sample preparation

Yogurt and drink yogurt samples were kindly provided by Mahdam Dairy Factory, 200 kilometers from Shiraz to Bushehr, Iran. For preparation of thyme‐added yogurt and thyme‐added drink yogurt samples, 5 ppm of EO from thyme was added to each of the yogurt and drink yogurt samples before filling and incubating.

#### Hydrodistillation

For extracting EO via hydrodistillation (HD) process, 25 g of the sample was submitted to water distillation in a Clevenger‐type apparatus with 500 mL water for 2.5 h at distillation rate of 3–3.5 mL min^−1^ according to the European Pharmacopoeia. After cooling, the oil was collected by use of a syringe. Dry sodium sulfate was added to remove water from the oil and after vigorous shaking and filtration, the EO was transferred into a brown, capped bottle, and stored in under refrigeration (Pharmacopoeia 1998).

#### Ultrasound‐assisted hydrodistillation

Ultrasound irradiation was applied by means of a Hielscher ultrasonic device (UP100H, 100 W, 30 kHz) equipped with a titanium sonotrode (tip diameter 10 mm) was used to sonicate the samples. The extraction unit was also equipped with an all glass clevenger‐type apparatus. The extraction process was performed by adding 25 g of the sample into 500 mL distilled water in a volumetric flask. The flask was then sonicated for 5 min which caused a 5°C temperature increase. Next, the sample was hydrodistillated for 80 min based on the HD procedure described above. After this period the temperature was decreased to 50°C and the second sonication process was performed for 5 min which caused a temperature increase in 6°C. Finally, the sample was hydrodistillated again for another 80 min. The EO was collected, dried under anhydrous sodium sulphate, and transferred into a brown, capped bottle and stored at 4°C until used (Palma and Barroso 2002; Hromadkova and Ebringerová 2003).

#### Ohmic extraction

The ohmic extractor unit consisted of a cylindrical Teflon chamber (0.07 m internal diameter and 0.25 m length) which was equipped with two Titanium electrodes. The system was fully automated for which the voltage (0–300 V) and current (0–16 A) and temperature could be controlled, monitored, and recorded to a data sheet throughout the experiment. In this extraction process 25 g of the sample was added to 500 mL brine (NaCl) solution (3 g L^−1^) and charged into the chamber (sodium chloride will provide sufficient electrical conductivity between two electrodes for the heat up process to be swift). The ohmic system was then switched on. A constant voltage of 250 V was applied between the two electrodes for 5 min boil the sample. The temperature rise was recorded at about 20°C min^−1^. After this period, the voltage was reduced to 120 V and kept constant for 25 min. Dry sodium sulfate was added to the extract and the purified EO was stored in sealed brown vials at refrigerator (Knirsch et al. 2010; Hashemi et al. 2011a; Lili et al. 2011).

#### Ultrasound‐assisted ohmic extraction

The extraction process was performed by adding 25 g of the sample to 500 mL brine (NaCl) solution (3 g L^−1^) in a volumetric flask and sonication for 5 min (100 W) by the cited ultrasonic device. Afterward, the flask's contents were poured into the chamber of ohmic extractor unit and after onset of boiling (250 V, 5 min), a constant voltage of 150 V was applied for 25 min. The EO was collected, dried under anhydrous sodium sulphate and transferred into a brown, capped bottle and stored in under refrigeration (Hashemi et al. 2011a).

### Gas chromatography‐mass spectrometry

The samples of thyme EO obtained by four different extraction methods were analyzed using gas chromatography–mass spectrometry (GC–MS). The analysis was carried out on a Agilent 6890 GC–MS instrument equipped with a HP‐5MS fused silica column (30 m long, 0.25 mm i.d., 0.25 mm film thickness); Helium was used as the carrier gas at a flow rate of 0.8 mL/min with a split ratio equal to 1:50 and 1 *μ*L of the obtained EO was injected. The injector and detector temperatures were kept at 250 and 280°C, respectively. The column temperature increased from 50 to 240°C at a rate of 3°C min^−1^ and from 240 to 300°C at a rate of 15°C min^−1^. The retention time at 50 and 300°C was 5 and 3 min, respectively. The mass ratio analyzed was 33 to 500 m z^−1^. The identification of compounds was based on retention times of n‐alkanes (C6–C24) that were injected after the oil at the same temperature and conditions. Compounds were identified by comparison of their RI with those reported in the literature and their mass spectrum was compared with the Wiley Library (Hudaib et al. 2002; Hashemi et al. 2011b).

### DPPH free radical scavenging activity

The radical scavenging capacity of EO from *Thymua danesis* for DPPH was monitored according to the method described by Burits and Bucar (Burits and Bucar 2000). Fifty microliters of different concentrations of the EO samples in methanol (10–60 mg/mL) were added to 5 mL of a 0.04 g L^−1^ methanol solution of DPPH. After a 30 min incubation period at room temperature under dark condition, the absorbance of the samples was read against a blank at 517 nm. Inhibition of free radical DPPH in percent (*I*%) was calculated in following way:

$$I\;\left(\%\right)=\left(A_{\mathrm{blank}}-A_{\mathrm{sample}}/A_{\mathrm{blank}}\right)\times 100$$

where *A*_blank_ is the absorbance of the control reaction (containing all reagent except the test compound), and *A*_sample_ is the absorbance of the test compound. EO concentration providing 50% inhibition (IC_50_) was calculated from the graph plotting inhibition percentage against EO concentration (Safaei‐Ghomi et al. 2009; Hashemi et al. 2011a).

### In vitro antimicrobial activity of the EO

Antibacterial activity of the EO against bacteria was assessed on *Escherichia coli* O157:H7 ATCC 35150 and *Staphylococcus aureus* ATCC 25923 by applying the standard disks diffusion technique. For reaching the stationary phase of growth, the bacterial cultures were activated 24 h before the onset of the assays and to reach the microorganism concentration of 10^5 ^CFU mL^−1^, Petri dishes from the cultures were inoculated with proper sterile media. An aliquot of dimethylsulfoxide (DMSO) was added to the EOs in order to obtain a 0.0235–0.75 mL mL^−1^ concentration range. After dropping sequential dilutions of the DMSO (700 g L^−1^)/EO solution on sterile paper disks they were placed on the center of the inoculated petri dishes. Accordingly, the petri dishes were incubated at 37°C for 24 h and subsequently were checked for the minimum inhibitory concentration (MIC) of the EO (Kacˇániová et al. 2012). The rest of experiments was performed by applying the EO which had the best in vitro antioxidant and antimicrobial properties.

### Antioxidant activity in sunflower oil

To determine the antioxidant properties of the EO, 3 g L^−1^ and 6 g L^−1^ of the EO were added to the sunflower oil and its effect on fatty acid composition of sunflower oil was compared to the control and native samples after a period of 4 days at 60°C. Gas chromatography (Agilent Technologies model 6890N, Germany) was used for fatty acid analysis (Lee et al. 2005).

### Antimicrobial activity of the EO in yogurt and drink yogurt samples

Pour plate technique was used for coliform counts and surface (spread) plate technique was done for yeast and mold counts (Mohammad and El‐Zubeir 2011). For enumeration of Total Yeast and Mold (TYM), and Coliform Bacteria, samples of yogurt and drink yogurt (10 g without and with 6 g L^−1^ EO) were dispersed in 90 mL sterile Ringer's solutions and appropriate decimal dilutions were prepared under the aseptic conditions. Yeast Extract Glucose Chloramphenicol Agar (YGC Agar) was used for TYM enumeration and plates were incubated at 25°C for 5 days. Coliform group bacteria were enumerated in Violet Red Bile Agar (Oxoid) after incubation at 37°C for 24 h (Karagozlu et al. 2008; Mohammad and El‐Zubeir 2011).

### Sensory analysis

Twelve trained panelists (six males and six females) were chosen from students of School of Agriculture of Shiraz University for the assessment of the sensory attributes of the yogurt and drink yogurt samples. In order to achieve similar conditions the samples were tempered at 4°C for 24 h before their sensory assessment. Each sample was coded using a three‐digit random number and served successively to the panelists in individually partitioned booths. Palatable attribute was used to rate the overall acceptability of the samples. Taste and odor evaluation was assessed by introducing five sensory numbering descriptors including: 1 to 5, wherein which number 5 presents the best quality. Panelists were motivated to express any criticisms on the score sheets used for the sensory evaluation.

### Experimental design and data analysis

For this research a completely randomized design (CRD) was used. The data were subjected to the analysis of variance (ANOVA) using SPSS (Abaus Concepts, Berkeley, California, U.S.A.) version 16.0. Significant differences between the means were determined using Duncan's new multiple range test at *α *< 0.05.

## Results and Discussion

### Antioxidant properties of the EO from *Thymua danesis*

#### DPPH test

As it is indicated in Table 1, the EO extracted by each of the four extraction methods has very good antiradical power and its IC_50_ is comparable to IC_50_ of BHT (18.2 ± 0.8 mg mL^−1^). The thyme EO antioxidant activity was depended on the extraction method and its antiradical power can be attributed to the phenolic compounds such as thymol and carvacrol which can scavenge the free radicals (Youdim et al. 2002).

**Table 1:** IC_50_ of the EO extracted by different extraction methods

Method of EO extraction	IC_50_ mg mL^−1^ (Mean ± SD)
HD	24.3^b^ ± 0.3*
UAHD	24.1^b^ ± 0.2^1^
OE	23.9^b^ ± 0.6
UAOE	27.8^a^ ± 0.7

*In this column different superscript letters indicate significant differences (*P* < 0.05).

^1^Each point is the average of three replicates.

#### In vitro Antimicrobial activity

As it is represented in Table 2 the minimum inhibitory concentration of the thyme EO for *Escherichia coli* and *Staphylococcus aureus* was depended on the extraction method. Thyme EO contains antibacterial compounds such as thymol and carvacrol which can prevent bacterial growth by making changes in ionic slope of the ion channels of bacterial cell walls (Burt 2004; Oussalah et al. 2007).

**Table 2:** MIC of the EO extracted by different extraction methods

Method of EO extraction	MIC mg mL^−1^ for *Escherichia coli* (Mean ± SD)	MIC mg mL^−1^ for *Staphylococcus aureus* (Mean ± SD)
HD	130.8^a^ ± 4.4*	117.8^a^ ± 4.6
UAHD	132.1^a^ ± 6.1^1^	118.2^a^ ± 5.2
OE	132.9^a^ ± 4.5	119.1^a^ ± 3.8
UAOE	125.5^b^ ± 5.6	110.3^b^ ± 6.1

*In each column different superscript letters indicate significant differences (*P* < 0.05).

^1^Each point is the average of three replicates.

It can be inferred from the information on Tables 1 and 2 that the thyme EO extracted by UAOE method had the best in vitro antioxidant and antimicrobial properties among the thyme extracts which were obtained by the other three cited extraction methods; therefore, the thyme EO was produced in bulk by UAOE method for performing the rest of the assays.

#### Effect of addition of the thyme EO on the stability of sunflower oil

Oxidation of sunflower oil results in alteration of fatty acid profile of the oil and these alterations monitored by GC (Table 3). To have a good perception of the effect of EO (extracted by UAOE method) added to sunflower oil samples (sample 1 = 3 g L^−1^ EO added and sample 2 = 6 g L^−1^ EO added), they were compared to native and control samples. By proceeding with oil oxidation, unsaturated fatty acids (especially linolenic acid and linoleic acid) were converted to saturated ones.

**Table 3:** Changes in fatty acid profile of sunflower oil after 4 days of storage at 60°C

Fatty acids	Native oil (Mean g L^−1^ ± SD)	Control (Mean g L^−1^ ± SD)	Sample 1 (Mean g L^−1^ ± SD)	Sample 2 (Mean g L^−1^ ± SD)
C14:0	0.11^a^ ± 0.03*	0.12^a^ ± 0.03	0.12^a^ ± 0.02	0.11^a^ ± 0.04
C16:0	9.43^b^ ± 0.21^1^	9.62^a^ ± 0.35	9.65^a^ ± 0.41	9.45^b^ ± 0.01
C16:1	0.07^a^ ± 0.01	0.10^a^ ± 0.23	0.10^a^ ± 0.44	0.08^a^ ± 0.03
C17:0	0.04^a^ ± 0.02	0.06^a^ ± 0.03	0.06^a^ ± 0.02	0.04^a^ ± 0.01
C18:0	3.33^b^ ± 0.13	4.03^a^ ± 0.22	4.00^a^ ± 0.19	3.35^b^ ± 0.13
T ‐C18:1	0^b^ ± 0.00	0.06^a^ ± 0.01	0.07^a^ ± 0.01	0.07^a^ ± 0.01
C18:1	23.80^b^ ± 0.62	24.83^a^ ± 0.41	24.85^a^ ± 0.59	23.81^b^ ± 0.38
Iso‐C18:1	1.75^a^ ± 0.20	1.18^b^ ± 0.16	1.25^c^ ± 0.15	1.72^a^ ± 0.19
T‐C18:2	0.25^c^ ± 0.01	0.43^a^ ± 0.01	0.37^b^ ± 0.01	0.25^c^ ± 0.01
C18:2	58.00^a^ ± 0.80	55.54^c^ ± 0.44	55.56^c^ ± 0.68	57.95^b^ ± 0.55
C20:0	0.20^b^ ± 0.02	0.31^a^ ± 0.04	0.30^a^ ± 0.02	0.22^b^ ± 0.01
T‐C18:3	0.18^b^ ± 0.01	0.28^a^ ± 0.01	0.24^a^ ± 0.01	0.19^b^ ± 0.01
C18:3	2.42^a^ ± 0.01	2.37^b^ ± 0.01	2.39^b^ ± 0.01	2.41^a^ ± 0.01
Iso‐C18:3	ND^b^	ND^b^	ND^b^	0.02^a^ ± 0.01
Ga‐C18:3	0.10^b^ ± 0.01	0.17^a^ ± 0.02	0.16^a^ ± 0.02	0.10^b^ ± 0.00
C22:0	0.23^b^ ± 0.03	0.72^a^ ± 0.05	0.69^a^ ± 0.04	0.23^b^ ± 0.04

ND, not detected.

*In each row different superscript letters indicate significant differences (*P* < 0.05).

^1^Each point is the average of three replicates.

It is clearly seen in Table 3 that addition of EO from *Thymua danesis* (especially at the level of 6 g L^−1^) to sunflower oil conserves its unsaturated fatty acids from oxidation.

### Antimicrobial activity of the EO in yogurt and drink yogurt samples

**Figure 1:**
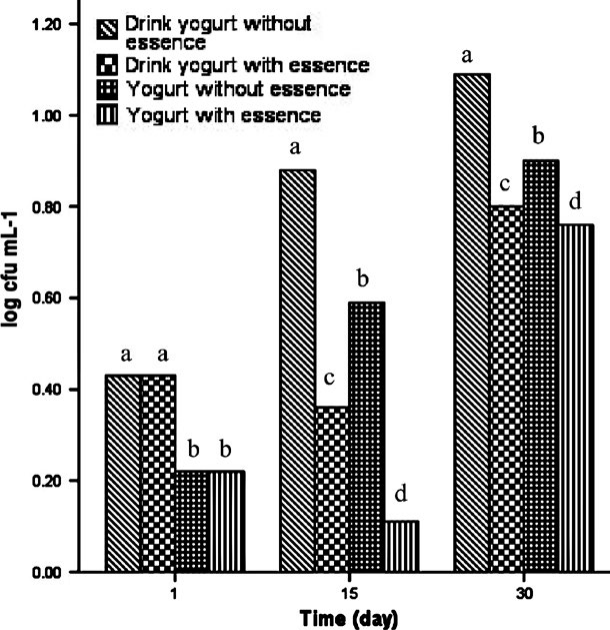
Antimicrobial activity of the EO in yogurt and drink yogurt samples against coliforms. In each column different letters indicate significant differences (*P* < 0.05).

**Figure 2:**
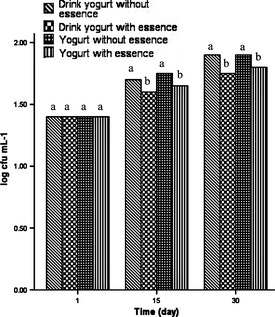
Antimicrobial activity of the EO in yogurt and drink yogurt samples against fungus. In each column different letters indicate significant differences (*P* < 0.05).

Adding 6 g L^−1^ thyme EO (extracted by UAOE method) to the yogurt and drink yogurt samples reduced the overall coliform and fungus counts during the admissible shelf life of these two products (30 days) to the below of their standard levels (10 cfu mL^−1^ for coliforms in each product and 100 cfu mL^−1^ for total yeasts and molds in each product). This antibacterial property which is ascribed to antibacterial compounds of thyme EO (like thymol and carvacrol) was not seen in the samples without EO and their total counts of coliforms and fungus exceeded from the standard level (Figs. 1, 2). The same results were cited in the Burt's review (Burits and Bucar 2000).

### Effect of adding thyme EO on the acidity and sensory properties of the yogurt and drink yogurt samples

**Figure 3:**
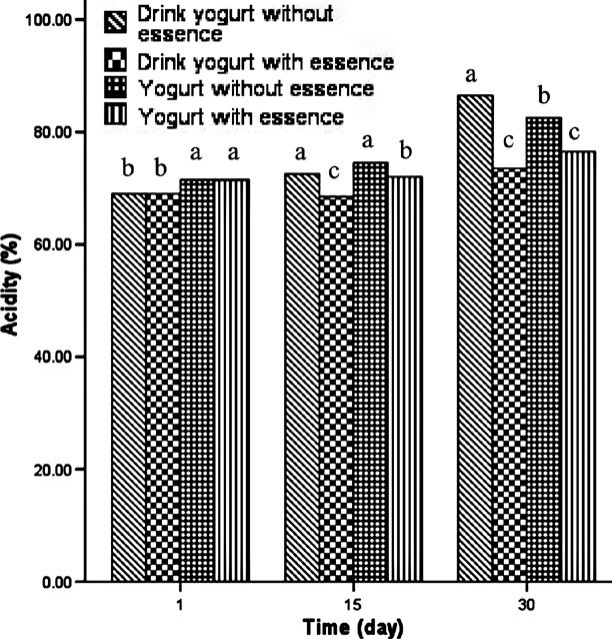
Effect of adding thyme EO on the acidity of the yogurt and drink yogurt samples. In each column different letters indicate significant differences (*P* < 0.05).

Figure 3 shows that during 30 days of storage at 4°C the acidity of the samples without EO was increased. However, in samples containing EO (extracted by UAOE method) their acidity remained relatively constant after 15 days of storage and thenceforward during the next 15 days of storage, the rate of increase in the acidity was less than the samples without EO. Prevention of acid production in the thyme EO added samples is ascribed to the antimicrobial properties of the thyme EO which suppresses the extra acid production by microorganisms.

**Figure 4:**
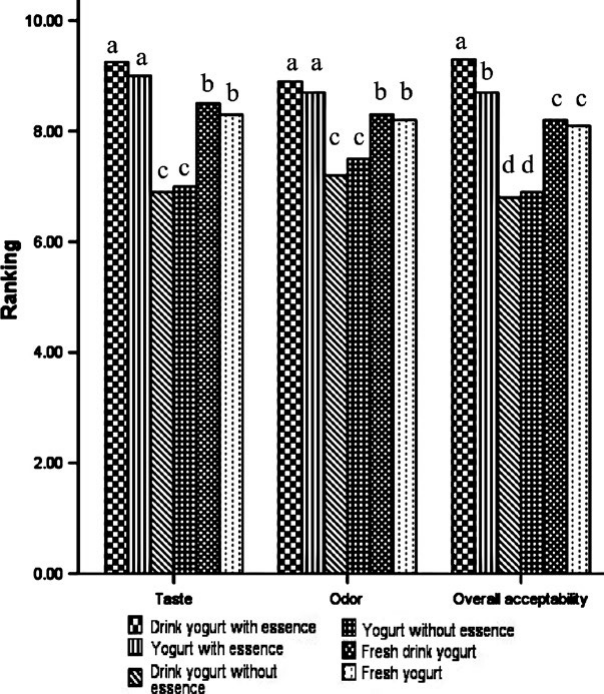
Effect of adding thyme EO on the sensory properties of the yogurt and drink yogurt samples. In each column different letters indicate significant differences (*P* < 0.05).

After 30 days of storage at 4°C, the thyme EO‐added and control (contained no EO but stored at 4°C for 30 days) samples, were served to panelists to compare them to fresh samples and evaluate their sensory properties. As it is observed in Figure 4 in all cases the samples containing thyme EO have the best taste, flavor, and overall acceptability among the other samples. This is mainly because of excellent sensory properties of thyme EO and controlling of extra acid production by limiting the bacterial growth.

## Conclusion

With regard to the excellent results of antioxidant, antibacterial, and sensory tests, it can be inferred that thyme EO can be used as an antioxidant, antimicrobial, and flavoring agent in food systems. Moreover, extraction method can influence the antioxidant and antimicrobial properties of the thyme EO, and in this view, UAOE method is the most effective method of extraction among the other three cited extraction methods.

## Conflict of Interest

None declared.
